# Exploring the Potential Mechanisms of *Melilotus officinalis* (L.) Pall. in Chronic Muscle Repair Patterns Using Single Cell Receptor-Ligand Marker Analysis and Molecular Dynamics Simulations

**DOI:** 10.1155/2022/9082576

**Published:** 2022-06-01

**Authors:** Yisheng Chen, Zhiwen Luo, Jinrong Lin, Beijie Qi, Yaying Sun, Fangqi Li, Chenyang Guo, Weiwei Lin, Xueran Kang, Xinyi He, Qian Wang, Shiyi Chen, Jiwu Chen

**Affiliations:** ^1^Department of Sports Medicine, Huashan Hospital, Fudan University, Shanghai, China; ^2^Department of Orthopedics, Shanghai General Hospital, Shanghai Jiao Tong University School of Medicine, Shanghai Jiao Tong University, Shanghai 200080, China; ^3^Department of Neurosurgery, Second Affiliated Hospital of Zhejiang University School of Medicine, Zhejiang University, 88 Jiefang Road, Hangzhou, 310009 Zhejiang, China; ^4^Shanghai Jiao Tong University, Shanghai 200080, China; ^5^State Key Laboratory of Genetics Engineering, Collaborative Innovation Center for Genetics and Development, School Life Sciences and Human Phenome Institute, Fudan University, Shanghai, China; ^6^Postdoctoral Workstation, Department of Central Laboratory, The Affiliated Taian City Central Hospital of Qingdao University, Taian 271000, China

## Abstract

Information regarding the function of *Melilotus officinalis* (L.) Pall. in skeletal muscles is still unknown. In this study, we explored the possible regulatory targets of M. (L.) Pall. that affects the repair patterns in chronic muscle injury. We analyzed the potential target genes and chemical composition of M. (L.) Pall. and constructed a “drug-component-disease target genes” network analysis. Five active ingredients and 87 corresponding targets were obtained. Muscle-tendon junction (MTJ) cells were used to perform receptor-ligand marker analysis using the CellphoneDB algorithm. Targets of M. (L.) Pall. were screened further for the cellular ligand-receptor protein action on MTJs. Enrichment analysis suggests that those protein-associated ligand receptors may be associated with a range of intercellular signaling pathways. Molecular docking validation was then performed. Five proteins (CCL2, VEGFA, MMP2, MET, and EGFR) may be regulated by the active ingredient luteolin and scoparone. Finally, molecular dynamics simulations revealed that luteolin can stably target binding to MMP2. M. (L.) Pall. influences skeletal muscle repair patterns by affecting the fibroblast interactions in the muscle-tendon junctions through the active ingredients luteolin and scoparone.

## 1. Introduction

*Melilotus officinalis* (L.) Pall. (M. (L.) Pall.) is a traditional Chinese medicine that is widely distributed and has broad prospects for development and utilization [1]. M. (L.) Pall. has antiedema, antioxidant, and hepatoprotective properties [1–3]. In ancient China, M. (L.) Pall. was used to treat a variety of chronic diseases [4]. M. (L.) Pall. is often used to reduce postoperative edema and promote early recovery after clinical orthopedic and sports medicine procedures. However, the function of M. (L.) Pall. in chronic skeletal muscle injury is unproven.

Skeletal muscle injuries are one of the most common sports injuries, accounting for approximately 40% of sports-related injuries in older people [5]. Muscles can be damaged by external forces, biological factors, and chemical factors [6, 7]. Excessive chronic injuries will lead to scar formation and fat infiltration [8, 9]. Therefore, understanding the factors influencing muscle repair can help promote skeletal muscle repair [10, 11]. Recent topical studies have explored the spatial-positional interactions of skeletal muscle regeneration and their underlying mechanisms to find new ways to improve the repair potential of skeletal muscles [12, 13]. Recent studies have found that the positional information driving limb muscle patterns are contained in the fibroblasts of the connective tissues [14]. Our previous studies have annotated and functionally analyzed these cells [15]. These cells have extensive intercellular interactions via the ligand-receptor pathway.

Liu et al. isolated 29 compounds from M. (L.) Pall. alcoholic extracts. They have good antioxidant activity and play an important role in anti-inflammatory and antioxidant functions [16]. Recent studies have also found that M. (L.) Pall. promotes wound repair [17]. This study provides a new way to explore these effects of M. (L.) Pall. in skeletal muscles by targeting the ligand-receptor pathway, which is important for drug function [18, 19]. Studying the effects of M. (L.) Pall. in the skeletal muscle fibroblasts allows us to explore its potential mechanisms in muscle repair patterns.

In this study, network pharmacology and molecular docking approaches have been used to predict the possible regulatory targets of M. (L.) Pall. in muscle repair patterns, to reveal the potential molecular mechanisms of this compound in regulating muscle repair patterns and provide new ideas for the treatment of skeletal muscle injury.

## 2. Materials and Methods

### 2.1. Screening for Active Ingredients and Targets of M. (L.) Pall.

The active ingredients of M. (L.) Pall. were obtained from previous research results [16]. The active ingredients of M. (L.) Pall. were screened from the Traditional Chinese Medicine Systems Pharmacology Database and Analysis Platform (TCMSP) [20] database using the following conditions: oral bioavailability (OB) ≥ 30% and drug − likeness (DL) ≥ 0.1820. Potential target genes of M. (L.) Pall. were obtained by converting the screened active ingredients into corresponding targets through the UniProt database (http://www.uniprot.org/).

### 2.2. “Drug-Component-Target” Network Construction

A “drug-component-target” network structure was constructed with the active ingredient and corresponding target genes of M. (L.) Pall. using the Cytoscape (version 3.7.2) software [21]. Each node and edge in the network was analyzed to determine the relationship between the diseases and drug actions.

### 2.3. Single-Cell Dataset-Based Receptor-Ligand Marker Analysis

Seurat results from our previous study of single-cell data analysis were used to perform ligand-receptor maker analysis [15]. Muscle-tendon junction (MTJ) cells were obtained from the GEO dataset of GSE168153 [14]. GSE168153 is a single-cell dataset describing fibroblasts in muscle tendon junctions. Receptor-ligand marker analysis of the MTJ cells was performed using the CellphoneDB algorithm (v2.1.2), to analyze the cellular interactions in MTJ regions [22]. After filtering with *P* < 0.05, key intercellular interactions were identified. The results were visualized using the dot_plot function in the CellphoneDB and the R software.

### 2.4. Screening and Molecular Docking Validation for the M. (L.) Pall. Cellular Action Targets in MTJs

R language and VennDiagram packages were used to obtain the M. (L.) Pall. targets on the MTJ cells [23]. The active ingredients of the drug were pretreated as shown in the following: screening of key targets and active ingredients in “drug-component-target,” downloading 3D structures of active ingredients (mol2 format) from PubChem database, hydrogenation, charge addition, root detection of ligands, search and definition of rotatable bonds, etc. [24]. The 3D structure of the target protein was downloaded from the Protein Data Bank, all hydrogen atoms were added, Gasteiger charges were calculated, and nonpolar hydrogens were combined and saved in the pdbqt format using the AutoDock software [25]. The parameter exhaustiveness was set to 20, and other parameters were set to default values. AutoDock Vina 1.1.2 was used for molecular docking, and PyMOL was used for plotting [26].

### 2.5. Molecular Dynamics Simulation

To perform molecular dynamics simulations (MD), the force field parameters of luteolin were generated in this study using ACPYPE Server, an online tool [25, 27, 28]. Protein force fields are described by CHARMM [29]. TIP3P is for water modeling. The simulation was performed after a slow increase in system temperature from 0 K to 307 K. MD simulations were performed using the GROMACS software under constant temperature and pressure conditions as shown in previous studies. Visualization of the results of molecular dynamics simulations was done using PyMOL [26, 29, 30].

### 2.6. Gene Ontology (GO) Functional Enrichment Analysis and Kyoto Encyclopedia of Genes and Genomes (KEGG) Pathway Enrichment Analysis

The study species was *homo sapiens*, and molecular function (MF), biological process (BP), and cellular component (CC) were used for GO enrichment analysis as previous researches [31–33]. The significance of the KEGG pathway was set at *P* < 0.05 to search for the major functional and in vivo pathways significantly enriched by the active ingredient targets. Bar graphs of the pathways in the GO and KEGG pathway enrichment analysis were plotted using the clusterProfiler toolkit in R and ggplot [19, 34].

### 2.7. Statistical Analysis

Statistical analyses were performed using the R software (version 3.6.3), and differences were significant at *P* < 0.05. Spearman's method was used for correlation analysis, and the results of the analysis were presented as chord plots using the circlize package of the R software (version 0.4.12) [35].

## 3. Results

### 3.1. Screening for Potentially Active Compounds in M. (L.) Pall.

**Figure 1:**
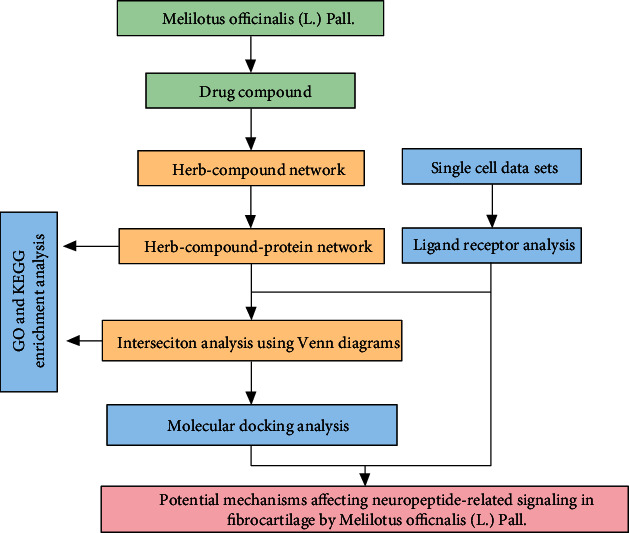
Research flow chart.

Figure 1 depicts the research flow of this study. The active ingredients of M. (L.) Pall. are based on previous research results [16]. Six active ingredients were screened according to the following conditions: OB ≥ 30% and DL ≥ 0.18 (Table 1).

### 3.2. Potential M. (L.) Pall. Targets of on the MTJ Cells

**Figure 2:**
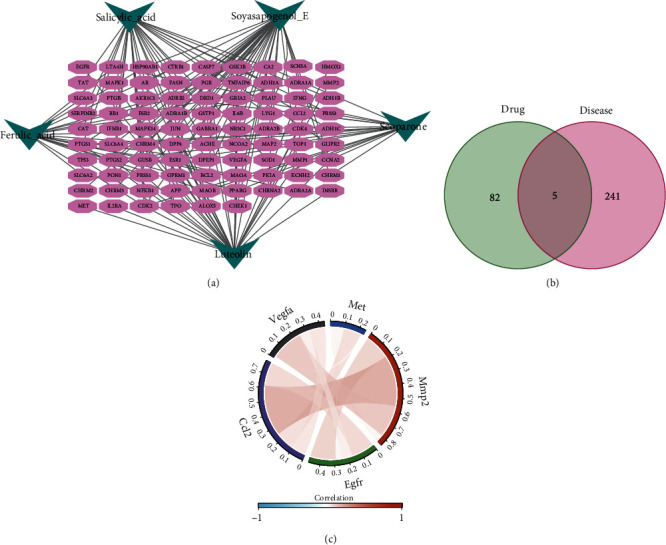
Potential role of M. (L.) Pall. (a) “drug-component-disease target genes” network; (b) drug target-MTJ ligand-receptor Venn diagram; (c) correlation analysis of five receptor-ligand-related genes (CCL2, EGFR, MMP2, MET, and VEGFA) demonstrated by the chord diagram.

Five of the six active ingredients had 87 corresponding targets (after excluding duplicates). The “herbal-active-component-disease target gene” regulatory network was constructed (Figure 2(a)). The red oval in the diagram represents the gene corresponding to the target protein. The green triangle represents the active ingredient. MTJ cells from the GSE168153 dataset were used for ligand-receptor analysis. All 246 potential ligand-receptor key genes were extracted. Cross-talk analysis revealed that M. (L.) Pall. might regulate the proteins of five genes (Figure 2(b)). Correlation analysis revealed that the expressions of CCL2, VEGFA, MET, MMP2, and EGFR were positively correlated with each other (Figure 2(c)).

### 3.3. Potential Effects of M. (L.) Pall. on MTJ Cells

**Figure 3:**
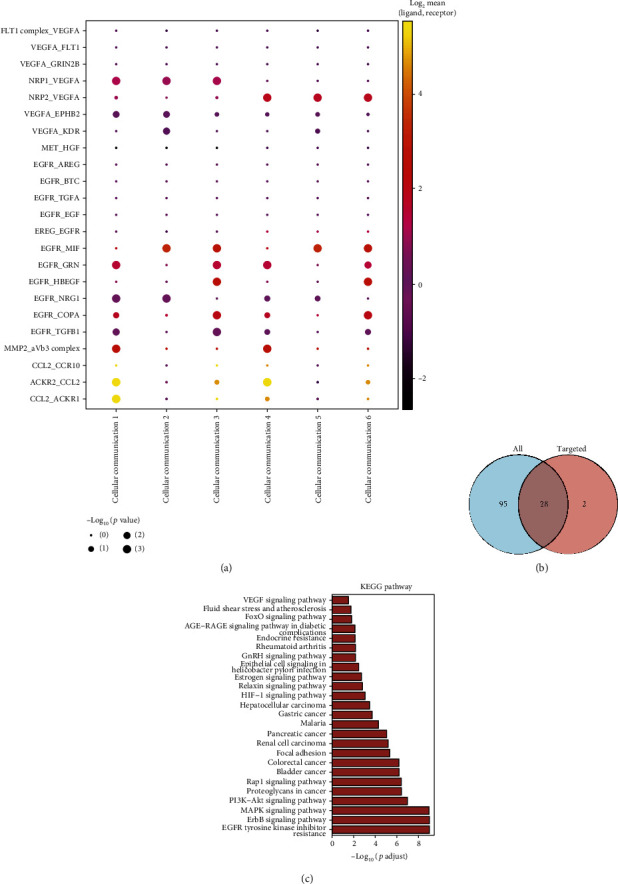
Ligand receptor analysis of MTJs targeted by M. (L.) Pall. (a) The key intercellular ligand-receptor interactions in MTJs, the vertical coordinate is the type of receptor-ligand linkage, and the horizontal coordinate is the corresponding cell-cell interaction; (b) “drug-component-disease target genes” network-MTJ ligand receptor-related pathway intersection analysis, “ALL” represents targets that M. (L.) Pall. may target, and “targeted” represents ligand-receptor-associated genes in (a); (c) enriched KEGG pathways.

**Figure 4:**
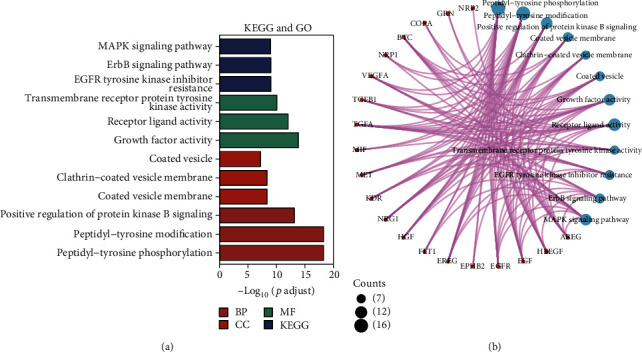
Enrichment analysis of core target proteins of M. (L.) Pall. (a) GO and KEGG enrichment analyses of core target proteins. (b) Distribution of the proteins in (a) from the GO and KEGG pathways.

As previously described, the data and cellular annotations for ligand-receptor maker analysis were obtained from GSE168153 database and our previous study, respectively. The receptor-ligand marker analysis of the MTJ cells was performed using the CellphoneDB algorithm (v2.1.2) (Figure 3(a)). The intersection analyses between the target enrichment pathways of M. (L.) Pall. and ligand receptor-related pathways of MTJs are shown in Figure 3(b). These target genes were found to be enriched in 28 KEGG pathways (EGFR tyrosine kinase inhibitor resistance, cancer-related pathway, epithelial cell signaling in *Helicobacter pylori* infection, rheumatoid arthritis, endocrine resistance; ErbB, MAPK, PI3K−Akt, Rap1, HIF−1, relaxin, estrogen, GnRH, and AGE−RAGE signaling pathways in diabetic complications; and so on). This suggests that M. (L.) Pall. may target some intercellular signaling pathways, such as neuropeptide-related, tumor-related, and stress direction-related pathways (Figures 2(b) and 2(c)). These genes were also enriched in metabolic functions (MF), including transmembrane receptor protein tyrosine kinase, receptor-ligand, and growth factor activities. Moreover, these genes were found to be enriched in cellular functions (CC), including coated vesicle, clathrin-coated vesicle membrane, and coated vesicle membrane. In biological processes (BP), these genes are enriched in the positive regulation of protein kinase B signaling, peptidyl-tyrosine modification, and peptidyl-tyrosine phosphorylation (Figures 4(a) and 4(b)).

### 3.4. Regulatory Relationship and Molecular Docking Analysis of Active Ingredient-Target Proteins of M. (L.) Pall.

**Figure 5:**
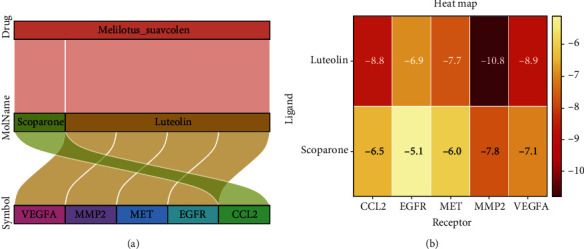
M. (L.) Pall. active ingredient-target protein regulatory relationships. (a) The Sankey diagram suggests that scoparone, the active ingredient of M. (L.) Pall. targets CCL2 and luteolin targets VEGFA, MMP2, MET, and EGFR. (b) Heat map showing the minimum binding energy of scoparone and luteolin to dock with target proteins CCL2, VEGFA, MMP2, MET, and EGFR.

**Figure 6:**
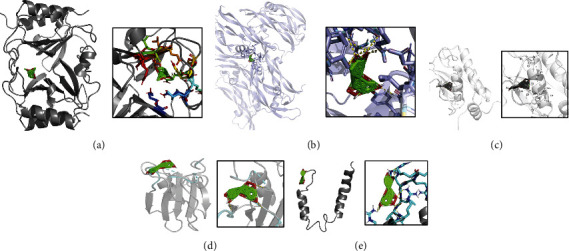
Schematic representation of molecular docking of target proteins with the active ingredients of M. (L.) Pall. (a) Interaction of scoparone with target CCL2 protein. (b) Interaction of luteolin with the target VEGFA protein. (c) Interaction of luteolin with the target MMP2 protein. (d) Interaction of luteolin with the target MET protein. (e) Interaction of luteolin with target EGFR protein. The dashed line indicates the hydrogen bond and marks the distance between the hydrogen bond and the compound.

Pharmacological database analysis suggests that scoparone, the active ingredient of M. (L.) Pall. targets CCL2 and luteolin targets VEGFA, MMP2, MET, and EGFR (Figure 5(a)). CCL2, VEGFA, MMP2, MET, and EGFR were molecularly docked to the two key pharmacodynamic components (scoparone, luteolin) of M. (L.) Pall. The lowest docking binding energy is shown in Figure 5(b). These molecular dockings are visualized in Figures 6(a)–6(e). The hydrogen bonds are indicated using dashed lines, and the distances between the hydrogen bonds and the compounds are also marked. The scoparone target binding CCL2 protein has a free energy of -6.5 kJ/mol. The free energies of luteolin target binding energy for VEGFA, MMP2, MET, and EGFR proteins are -8.9, -10.8, -7.7, and -6.9 kJ/mol, respectively. The minimum binding energies of these two key components to CCL2, VEGFA, MMP2, MET, and EGFR were less than -5.0 kJ/mol suggesting that M. (L.) Pall. exerts its effects mainly by targeting these components through scoparone and luteolin.

### 3.5. Molecular Dynamics Simulations of MMP2 and Luteolin

**Figure 7:**
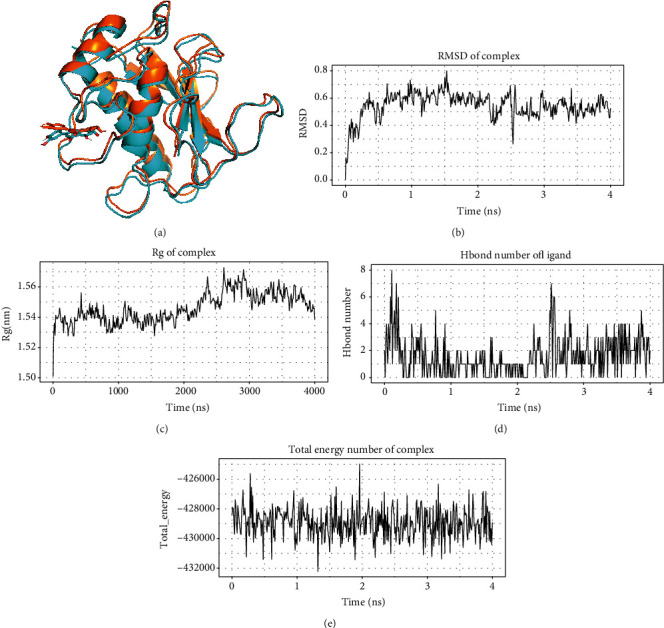
Molecular dynamics simulations of MMP2 and luteolin. (a) Conformational changes of the complex before and after MD, brown indicates before MD, and blue indicates after MD. (b) Variation of the root mean square displacement (RMSD) of the MMP2-lutelin complex. (c) Variation of the radius of gyration (Rg) of the MMP2-lutelin complex with time. (d) Variation of the number of hydrogen bonds formed between MMP2 and luteolin with time. (e) The free energy of binding between the small molecule ligand and the protein over time.

In the above molecular docking analysis, MMP2 and luteolin had the minimum binding free energy. The free energy of luteolin target binding energy for MMP2 is -10.8 kJ/mol. To further assess the binding efficacy of MMP2 and luteolin, MD simulations were performed (Figure 7(a)). Due to the interaction between MMP2 and luteolin, the root mean square displacement (RMSD) was found to increase at first and then stabilise (Figure 7(b)). The radius of gyration (Rg) of the MMP2-lutelin complex was also found to stabilise with the time passing (Figure 7(c)). In addition, the number of hydrogen bonds formed by MMP2 with luteolin remained in a relatively stable range (Figure 7(d)). The overall free energy in the system was also found to be stable (Figure 7(e)). Finally, MD simulations revealed that luteolin can stably target binding to MMP2.

## 4. Discussion

To the best of our knowledge, this study is the first to assess the impact of M. (L.) Pall. on the musculotendinous junction. According to the TCMSP database, M. (L.) Pall. has five active ingredients (OB ≥ 30% and DL ≥ 0.18) potentially acting on 87 targets. Therefore, the “herb-active ingredient-disease target gene” network was constructed. We found that M. (L.) Pall. influences the interaction of fibroblasts in muscle-tendon junctions and affects muscle repair patterns through the modulation of five ligand receptor-related proteins (CCL2, VEGFA, MMP2, MET, and EGFR) using the active ingredients luteolin and scoparone. And MD simulations revealed that luteolin can stably target binding to MMP2.

Scoparone is a naturally occurring coumarin found in green plants. It is purified from a lipid-lowering herb that reduces the proliferation of human peripheral blood mononuclear cells, scavenges reactive oxygen species, inhibits tyrosine kinase, and enhances the production of prostaglandins [36]. Recent studies have confirmed that scoparone has various biological activities such as antifibrosis, antioxidant, and fat differentiation inhibition [37–39]. Scoparone was also found to inhibit high-glucose-induced activation of the ERK1/2 signaling pathway in thylakoid cells and played an active role in inhibiting the accumulation of extracellular matrix in the high-glucose microenvironment [40]. In this study, scoparone was found to target CCL2. CCL2 is a ligand for CCR2. Inhibition of CCR2 after injury promotes skeletal muscle regeneration and function recovery [41, 42]. Our study suggests that scoparone may inhibit the function of CCR2 by binding to CCL2, and this may be a potential mechanism for M. (L.) Pall. to promote skeletal muscle regeneration.

Recent studies have found that luteolin protects skeletal muscles from attrition caused by inflammation and downregulates the expression of proteins associated with muscle catabolism [43]. Luteolin increased muscle strength in fatigued subjects and improved skeletal muscle contraction during ischemia and reperfusion [44]. In this study, luteolin targeted VEGFA, MMP2, MET, and EGFR. Luteolin inhibits VEGFA and affects microvascular networks formed during neovascularization in mice [45]. In tumors, luteolin inhibits MMP2 and MET [46–48]. Luteolin also inhibits downstream signaling molecules activated by EGFR, especially the Akt and MAPK signaling pathways [49].

Luteolin was found to target MMP2 in MD simulations. Elevated levels of MMP2 expression are associated with insulin resistance due to extracellular matrix (ECM) remodelling in skeletal muscle [50]. This suggests that luteolin targeting of MMP2 may have the potential to improve insulin resistance due to ECM. MMP2 has been found to be widespread in skeletal muscle, and therefore, the study of its function is important for exploring the repair of skeletal muscle after injury [51]. For example, both exogenous hydrogen sulphide and electroacupuncture treatments can improve skeletal muscle injury and reduce skeletal muscle fibrosis by downregulating MMP2 and related pathways [5, 52]. In the present study, luteolin was found to target MMP2, which may contribute to skeletal muscle injury repair.

This study provides new data supporting the treatment of sports injuries using M. (L.) Pall. and provides a theoretical basis for clinical application. All the core components of M. (L.) Pall. were screened and docked successfully with their key targets. The core components of M. (L.) Pall. have good binding activities to their key targets, suggesting that they can effectively treat skeletal muscles. However, more Chinese medicine databases need to be used, and target prediction databases need to be improved. This study did not directly examine the chronic muscle injury dataset, but rather by examining the MTJ fibroblastic dataset, which may be biased. Furthermore, more in vivo and ex vivo clinical studies are also needed to validate the mechanisms of action M. (L.) Pall. in the treatment of chronic skeletal muscle injury.

## 5. Conclusion

In summary, this study demonstrates that M. (L.) Pall. can skeletal muscle injury by acting on CCL2, VEGFA, MMP2, MET, and EGFR, through luteolin and scoparone. They are the key active ingredients of M. (L.) Pall. and affect intercellular signaling, such as neuropeptide-related, tumor-related, and stress-related pathways. M. (L.) Pall. may influence the interaction of fibroblasts in muscle tendon junctions to affect muscle repair patterns. Molecular docking analysis validated some of the network pharmacology results and confirmed the multicomponent, multitarget, and multipathway characteristics of M. (L.) Pall. in the treatment of skeletal muscle injury.

## Figures and Tables

**Table 1:** Six active ingredients of M. (L.) Pall. were screened.

Molecule name	Molecule ID	OD	Drug-likeness
Scoparone	MOL001999	74.75	0.09
Ferulic acid	MOL000360	39.56	0.06
Soyasapogenol E	MOL003651	37.64	0.75
Beta-sitosterol	MOL000358	36.91	0.75
Luteolin	MOL000006	36.16	0.25
Salicylic acid	MOL001801	32.13	0.03

## Data Availability

Muscle tendon junction (MTJs) cells are from the GEO dataset in the GSE168153 database.
